# Assessing Nursing Students’ Readiness to Address Sexual Health: Psychometric and A Mixed-Method Approach

**DOI:** 10.3390/nursrep16020072

**Published:** 2026-02-18

**Authors:** Nina Brkić-Jovanović, Bojana Tankosić, Jelena Lukić, Dragana Simin, Dragana Milutinović

**Affiliations:** 1 Department of Psychology, Faculty of Medicine, University of Novi Sad, 21000 Novi Sad, Serbia; 2 Department of Psychology, Faculty of Philosophy, University of Novi Sad, 21000 Novi Sad, Serbia; g.jelena85@gmail.com; 3 Department of Nursing, Faculty of Medicine, University of Novi Sad, 21000 Novi Sad, Serbia; dragana.simin@mf.uns.ac.rs (D.S.); dragana.milutinovic@mf.uns.ac.rs (D.M.)

**Keywords:** sexual health, nursing students, education, SA-SH-Ext, mixed-methods research

## Abstract

**Background/Objectives:** Sexual health is a crucial yet often overlooked aspect of nursing care, and nursing students often lack the communication skills needed to discuss it. Although several instruments are available to evaluate students’ attitudes and barriers, evidence on culturally adapted tools for Serbian nursing students remains limited. Therefore, the study aimed to assess the psychometric properties of the Serbian version of the SA-SH-Ext and to explore nursing students’ attitudes and barriers to sexual health communication. **Methods:** A sequential mixed-methods design was used. A total of 180 nursing students completed the SA-SH-Ext and SABS scales, followed by psychometric analysis including exploratory and confirmatory factor analyses and reliability testing. Semi-structured interviews with 20 students were thematically analysed to explore experiences and communication challenges. **Results:** Factor analysis yielded a four-factor model with the factors Being Comfortable, Communication with People with Disabilities, Future Patient and Working Relations, and Education and Competence, which explained 60.6% of the variance. The scale demonstrated strong internal consistency. Male and younger students reported higher comfort levels. Qualitative findings revealed discomfort, limited training, and fear of patient reactions, especially when discussing sexual health with older, disabled, or terminally ill patients. **Conclusions:** The Serbian SA-SH-Ext is a valid and reliable tool for assessing readiness to address sexual health. Despite positive attitudes, students face significant barriers. Integrating structured education into nursing curricula is essential to building competence and reducing stigma around sexual health in clinical practice.

## 1. Introduction

Sexual health is a crucial yet often overlooked aspect of nursing care. The World Health Organisation (WHO) defines it as a state of physical, emotional, mental, and social well-being, encompassing not only the absence of disease but also the presence of optimal health [1]. Despite its significance, cultural norms and systemic barriers frequently hinder discussions, particularly in conservative societies [2]. Nursing professionals struggle to address sexual health due to personal discomfort, lack of training, and sociocultural influences [3]. Studies indicate that students often lack the communication skills needed to discuss sexual health [4,5]. European research shows that many students consider sexuality a private matter, leading to limited discussions [4]. In Norway, cultural taboos and inadequate training reinforce these challenges [6].

Cultural attitudes significantly impact students’ willingness to engage in sexual health discussions. In Turkey, nursing students feel uncomfortable addressing sexual topics with patients due to religious and societal beliefs [7]. Similarly, Serbian nursing students acknowledge the importance of sexual health but struggle with conservative social norms and insufficient training [8,9]. In contrast, U.S. studies report a more positive outlook, although gaps in training remain [10].

The Students’ Attitudes toward Addressing Sexual Health (SA-SH) questionnaire, developed by Areskoug-Josefsson et al. [11], measures students’ confidence in discussing sexual health. It evaluates comfort in communication, perception of the work environment, and fear of negative patient interactions. The tool has demonstrated strong validity and has been adapted for use in various educational settings [12,13]. The Danish adaptation (SA-SH-D) has been validated as a reliable tool for assessing students’ attitudes toward sexual health [14,15]. Similarly, the Portuguese version has proven effective in identifying cultural and educational gaps [16].

An extended version, SA-SH-Ext, includes additional items that assess students’ confidence in working with diverse patient populations, such as individuals with disabilities [12]. Research by Ślusarska and Marcinowicz [17] identified two further dimensions: Educational Needs—Awareness of the Knowledge Gap and Educational Needs—Awareness of Competencies.

While tools like SA-SH-Ext provide valuable insights, they do not fully capture the complexities of barriers to sexual health discussions. Research highlights the need for structured, culturally sensitive educational programs to equip students with essential communication skills [14]. Mixed-method research, combining quantitative and qualitative data, can help refine curricula and address educational deficiencies.

Akalin and Ozkan [18] emphasise that sexuality-related courses should be introduced early in nursing education to overcome barriers. Similarly, it was revealed that students from conservative backgrounds became more open to discussing sexuality through structured education [19]. These findings underscore the importance of interactive learning approaches that encourage open dialogue on sexual health.

This study aims to examine nursing students’ attitudes toward discussing sexual health using a mixed-method approach. In this context, readiness refers to students’ willingness and perceived preparedness to address sexual health in clinical interactions, attitudes reflect personal beliefs and comfort regarding such discussions, while communicative competence relates to the skills required to initiate and conduct conversations about sensitive sexual health issues with patients. Key research questions include the following:

- What are the psychometric properties of SA-SH-Ext in Serbian students?
- What are nursing students’ attitudes toward addressing sexual health?
- What are the perceived barriers to addressing sexual health in clinical practice?
- What educational supports are needed to enhance students’ readiness for sexual health communication?

By addressing these questions, the study contributes to a deeper understanding of how nursing education can be restructured to support comprehensive, inclusive, and culturally sensitive patient care.

## 2. Materials and Methods

### 2.1. Study Design and Setting

The study employed a mixed-methods research design. The quantitative study part was conducted to assess the psychometric characteristics of the SA-SH-Ext and to estimate nursing students’ attitudes and beliefs regarding the addressing of sexual health.

This part of the descriptive, analytical, comparative, and psychometric study was conducted at the Department of Nursing, Faculty of Medicine of the University of Novi Sad, Serbia, during the summer semester of the 2023/2024 academic year. The second step was followed by qualitative data collection. To investigate in-depth, real-life experiences, attitudes, and beliefs and confirm the quantitative findings, 20 nursing students, four from each academic year, were interviewed using a semi-structured interview technique. The study adhered to the Consolidated Criteria for Reporting Qualitative Studies (COREQ) [20] and the Systematic Development of Standards for Mixed Methods Reporting in Rehabilitation Health Sciences Research (MMR-RHS) guidelines [21].

### 2.2. Sample and Data Collection

For the quantitative segment of the study, using convenience sampling, 208 out of 240 (86.66%) questionnaires were received. However, among the total received questionnaires, 28 were excluded due to missing values, resulting in a total effective sample size of 180 for the study.

The minimum required sample size was calculated using a standard formula for sample size estimation for finite populations, assuming a 95% confidence level and a 5% margin of error. Based on this calculation, the required minimum sample size was 150 participants. The final effective sample size of 180 students, therefore, exceeded the calculated requirement, indicating that the study was adequately powered to provide reliable and representative quantitative results for the target population.

The sample consisted of 23 males (12.8%), 157 females (87.2%), and 2 students of unknown sex (1.1%). Students were also categorised based on study year: 52 students participated from the first year (28.9%), 61 students from the second year (33.9%), 38 students from the third year (21.1%), 22 from the fourth year (12.2%), and seven students (3.9%) from the master’s program (5 years). Students also varied by religion, with the majority identifying as Orthodox (146, 81.1%), followed by Catholic (13, 7.2%), Protestant (6, 3.3%), and a mixed group of other religions (15, 8.4%).

In the quantitative part of the study, data were collected via paper questionnaires distributed in sealed envelopes in the classroom after regular lectures. The study was introduced by a faculty member who was not involved in nursing teaching program or assessing the participating students and had no prior academic relationship with them, thereby reducing the potential for perceived academic pressure. Participation was entirely voluntary, and students were clearly informed that their decision to participate or decline would have no impact on their academic standing, course requirements, or relationship with the institution. Data collection took place outside of any evaluative context, and informed consent was obtained from all participants prior to inclusion in the study. Students self-selected into the study by choosing to complete the questionnaire and, for the qualitative component, by expressing willingness to participate in an interview after receiving an email invitation to participate.

Before filling out the questionnaire, the students were informed about the study, and on the first page of the questionnaire, there was a brief introduction about sexual health as an essential segment of general health and quality of life, along with the definition of sexual health given by the World Health Organisation [1].

For the qualitative segment of the study, 20 students were interviewed by the same interviewer (Appendix A), with 4 students from each study year recruited purposively. The purposive sampling method was employed in this study to gain an in-depth understanding of and description of a particular group [22,23]. A pilot test of the protocol and a cognitive interview were conducted with two students who did not participate in the main study to verify the procedure. The interviews lasted an average of 34 min, were audio-recorded, and the interviewer took notes during the conversations. The number of students in each group allowed the differences identified in the analysis to represent group differences rather than individual variation. These 20 students belonged to different years of study, genders, and religions. The issues of anonymity and confidentiality for students in qualitative research were discussed prior to the commencement of the study.

The interview protocol guide included the following main areas:

1. Students’ knowledge about the concept of sexuality and how it is discussed in the general population.
2. Bringing up sexuality issues with patients.
3. Addressing sexuality issues with patients concerning some of their characteristics.
4. Who should be responsible for patient sexuality issues in the healthcare team?
5. The extent of education on the topic and its importance for the patient’s complete psychophysical recovery.

### 2.3. Student-Reported Measures for the Quantitative Part of the Study

The general questionnaire for obtaining sociodemographic data, an extended version of the Students’ Attitude Towards Addressing Sexual Health (SA-SH-Ext), and the Sexual Attitude and Beliefs Survey (SABS) were used as measures of students’ reports. The general questionnaire included four items to obtain the following data: gender, study year, religion, and sexual orientation.

The SA-SH-Ext contains 27 items grouped into four domains: Being Comfortable (items 1–12), Future Patient Relations (items 13–19), Future Relations with Colleagues (items 20–22), and Education and Competence (items 23–27). All items were assessed on a 5-point Likert scale, with responses coded from 1 (most negative) to 5 (most positive). Items 13 to 18 and 20 to 22 are negatively worded and reversed for analysis. The total score is calculated by summing the answers to all items, with a possible range from 27 to 135 [13].

The SABS contains 12 items rated on a 1–6 Likert scale, where 1 indicates “strongly disagree”, and 6 indicates “strongly agree.” The questionnaire is one-dimensional, with a theoretical range of scores from 12 to 72, where a higher score indicates greater obstacles in assessing patient sexuality and providing sexual health counselling in nursing practice. Seven items were reverse-scored to avoid response bias [24]. In this study, the SABS demonstrated a one-factor structure with a Cronbach alpha coefficient of 0.759, which was used as a correlate of construct validity.

### 2.4. Data Analysis

Statistical data analysis was performed using Jamovi 2.3.26 (Jamovi, 2022) and the IBM SPSS Statistics 26.0 package (IBM Corp., New York, NY, USA, 2019). Numerical characteristics are presented using mean values (arithmetic mean ± SD), and attributive characteristics are presented using frequencies and percentages. Univariate analysis of domain scores across sociodemographic factors was performed using independent *t*-tests and one-way analysis of variance (ANOVA) with LSD post hoc analysis or suitable non-parametric substitutions if the sample sizes did not permit parametric analyses. Pearson’s r was used to assess the association between the two instruments and their domains.

Confirmatory factor analysis and the Cronbach alpha coefficient were used to check the structural validity and reliability of the instruments. The following indices were used: the penalising function (χ^2^/degrees of freedom [df]), with values lower than 3 indicating good fit; the comparative fit index (CFI), which ranges from 0 to 1 with a minimum good fit value of 0.90; the standardised root mean square residual (SRMR) index, with values lower than 0.08; and RMSEA < 0.05 indicating good fit. In addition, convergent validity and construct reliability were evaluated by calculating Average Variance Extracted (AVE) and Composite Reliability (CR) based on standardized factor loadings obtained in confirmatory factor analysis. To estimate the test–retest reliability of the SA-SH-Ext, intraclass correlation coefficients (ICCs) with 95% confidence intervals (CIs) were calculated. Bartlett’s test of sphericity and the Kaiser–Meyer–Olkin (KMO) test were used to assess sampling adequacy and data factorability.

When the initially tested confirmatory model did not demonstrate satisfactory fit, exploratory factor analysis (EFA) was subsequently performed to identify a factor structure better suited to the data. Factor retention was guided by eigenvalues, scree plot inspection, and interpretability of the factor solution. During exploratory factor analysis, item retention was guided by established psychometric criteria. Items were considered for removal if they demonstrated: (1) factor loadings below 0.40, (2) cross-loadings exceeding 0.30 on more than one factor, (3) low communalities (<0.30), or (4) lower alpha if the item is not deleted. Item removal was performed iteratively to achieve a parsimonious and interpretable factor solution while maintaining the construct’s theoretical coherence.

For the qualitative study, results were analysed using a grounded, multi-stage approach [25,26]. With the students’ consent, all interviews were recorded, transcribed verbatim, and subjected to thematic analysis using Braun and Clarke’s six-phase guide [27]. Before undertaking the analyses, the students were allowed to review the transcripts. The transcripts were imported into MAXQDA (VERBI Software, 2018), and three researchers independently identified, reviewed, and labelled themes to ensure reliability [28]. Data collection and analysis were conducted concurrently, and data saturation was considered achieved when no substantially new themes emerged in the final interviews. Coding was performed independently by three researchers, followed by discussion until consensus was reached. Methodological triangulation was achieved by integrating quantitative and qualitative findings within the mixed-method design, while investigator triangulation was ensured through the involvement of researchers from different disciplinary backgrounds, enhancing the credibility and consistency of the findings. A summary of the results was sent to the students as feedback.

### 2.5. Ethical Approval

The study was conducted in accordance with the principles of ethical research involving human subjects. This manuscript reports findings from one component of a larger multi-part study. The overarching study was designed to address multiple methodologically distinct research objectives; therefore, the findings were reported across separate publications to ensure adequate analytical depth and methodological transparency. Ethical approval for the entire study was obtained from the Faculty of Medicine Research Ethics Commission, University of Novi Sad, Serbia (Ref. No. 01-39/12/1; approval date: 31 January 2023). Before data collection, all eligible survey and interview students received a written statement explaining the purpose of the study, the anonymous and voluntary nature of participation, and a guarantee that (non)participation would not affect their further education.

During qualitative data collection, the researcher verbally shared the study’s aims and objectives with the students in their native language (Serbian).

## 3. Results

### 3.1. Psychometric Analysis of the SA-SH-Ext

Bartlett’s test of sphericity indicates that the correlation results are not random (χ^2^ = 2924.815, *p* < 0.001). The KMO test, with a value of 0.876, suggested that the sample was adequate for the variables in the model and the model as a whole, satisfying the conditions for conducting the factor analysis of the questionnaire.

The initially assumed four-factor model did not give a good model fit (χ^2^ = 901, *p* < 0.05, CFI = 0.78, RMSEA = 0.101, TLI = 0.76, percentage of variance 48.1%); with exploratory factor analysis, we also obtained a significant four-factor model with items somewhat differently grouped by factors (Table 1). However, the interpretation of model fit should be made with caution, as some indices do not fully meet commonly recommended thresholds. The results of this four-factor model indicated a marginally acceptable model fit (χ^2^ = 274, *p* < 0.001, CFI = 0.91, RMSEA = 0.09, TLI = 0.85). In particular, the RMSEA value of 0.09 is at the upper boundary of acceptable fit according to more recent methodological recommendations [29]. The percentage of explained variance with these four factors is 60.6%. Items 10, 13, 14, 17, 18, 19, 22, and 23 were excluded from further analysis because they demonstrated low factor loadings, cross-loadings across multiple factors, or low communalities, which reduced clarity and interpretability of the factor structure. Their removal resulted in a more stable and theoretically coherent four-factor solution, with improved factor interpretability and increased explained variance. The final version, therefore, retained 19 items distributed across four factors.

The first extracted factor, *Being Comfortable*, refers to how comfortable students feel communicating about sexuality with patients and includes questions 1, 2, 3, 4, 9, 11, and 12, accounting for 24.3% of the variance. The second factor, which explains 14.9% of the variance, was named *Communication with People with Disabilities* because it includes questions 5, 6, 7, and 8, which emphasise the possibility of communication about sexuality with people with physical and mental disabilities or illnesses. The third factor was titled ‘*Future Patient and Working Relations*’ because it grouped questions 15, 16, 20, and 21, which address the students’ attitudes regarding how patients and future colleagues may feel uncomfortable discussing topics related to sexuality. It explains 10.7% of the variance. The fourth factor, named *Education and Competence*, refers to the student’s perception of having adequate knowledge, skills, and competence to address sexuality in communication with patients in future work and includes questions 24, 25, 26, and 27. The percentage of variance it explains is 10.8.

The reliability of this scale was assessed using Cronbach’s alpha. The reliability of all four subscales ranges from satisfactory to exceptional: for the first subscale, Being Comfortable, it is 0.94; for the second subscale, Communication with People with Disabilities, it is 0.91; for the third subscale, Future Patient and Working Relations, it is 0.82; and for the fourth subscale, Education and Competence, it is 0.77. Composite reliability values ranged from 0.76 to 0.89, confirming satisfactory internal consistency across all four factors. Average variance extracted values ranged from 0.44 to 0.56, indicating acceptable convergent validity overall, although slightly lower AVE values were observed for two factors (Future Patient and Working Relations and Education and Competence).

According to the COSMIN guidelines [30], two random groups of nursing students were formed. There were ten students per group, and the similarity of their answers to a particular item was tested. The average correlation between the two groups of students’ answers was 0.59, indicating moderate measurement invariance.

Test–retest reliability was assessed in a sample of 15 students randomly selected from the total sample, with a 4-week interval between the two tests. It was recommended to be sufficiently long to minimise recall bias while being short enough to reduce the effect of maturity. The interclass correlation coefficient was excellent, with a value of 0.915 (95% CI = 0.902–0.965).

The standard measurement error for each subscale indicates a small range of potential answers and a low possibility of error: for *Being Comfortable*, it was 0.69; for the subscale *Communication with People with Disabilities*, it was 0.29; for *Future Patient and Working Relations*, it was 0.19; and for the subscale *Education and Competence*, it was 0.23.

The correlations between variables are presented in Table 2. All four subscales of the SA-SH-Ext correlate significantly and strongly negatively with the SABS questionnaire score at the *p* < 0.001 level. All subscales of the SA-SH-Ext correlate with each other, with significant low-to-strong positive correlations (Table 2).

### 3.2. The Factors Associated with Being Comfortable, Communication with People with Disabilities, Future Patient and Working Relations, and Education and Competence

Univariate analysis of the factors associated with *Being Comfortable*, *Communication with People with Disabilities*, *Future Patient and Working Relations*, and *Education and Competence* are presented in Table 3. In the Being Comfortable domain, additional exploratory findings suggest differences across gender and year of study, with higher scores observed among male students and master’s students. Although first-year students also showed higher scores than fourth-year students, these results should be interpreted with caution due to insufficient statistical power within subgroups and require confirmation in future studies with larger, adequately powered samples.

In the *Future Patient and Working Relations* domain, additional exploratory differences related to sexual orientation and year of study were observed, with lower scores among master’s degree students and students with homosexual orientation. Given the limited sample size and sampling strategy, these findings are considered preliminary and indicative rather than confirmatory.

In the domains of Communication with People with Disabilities and Education and Competence, no differences were observed in scores concerning sociodemographic variables.

### 3.3. Qualitative Analysis

**Figure 1:**
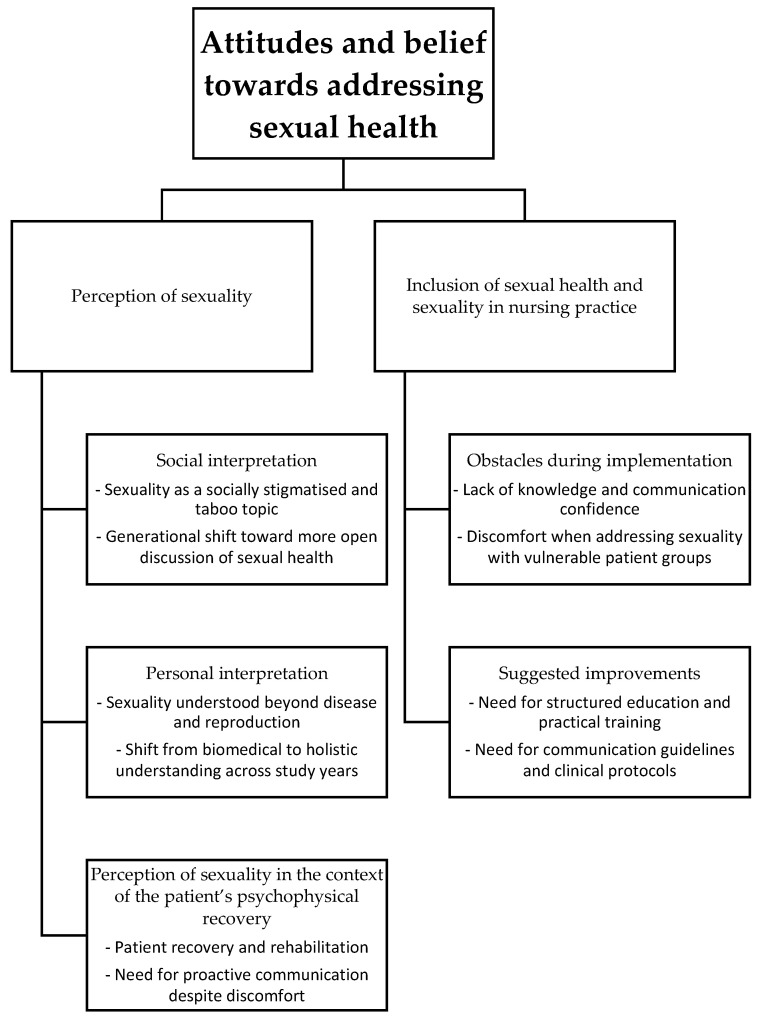
The two main themes and subthemes.

Data analysis revealed two main themes (Figure 1). The findings are presented and supported by indicative quotes from students.

#### 3.3.1. Theme 1: Perception of Sexuality

**Social interpretation**

The first theme explored the nursing students’ beliefs regarding sexual health in the context of nursing care. Nursing students, in the majority, estimate that opening topics related to sexuality, on different levels, is socially stigmatised, including in the nursing care context. The presence of generational differences actively maintains the process of stigmatisation, that is, traditional and/or patriarchal beliefs, pointing to the social aspect of sexuality, which is influenced by laws, taboos, pressures, and resistances within families, that is, society as a whole.

*“Sexuality is still a taboo topic here. And further. As a people, we are traditional, and somehow, as if we are trying to cover it up, very few of us find the strength and courage. Moreover, that should be talked about”* (first-year student).

The shift from traditional to more “modern,” open approaches to sexuality among students in the final year of undergraduate and master’s academic studies is reflected in greater openness when discussing sexual health topics, a stronger emphasis on patient-centred communication, and a reduced reliance on traditional norms.

**Personal interpretation**

Personal interpretations of students concerning topics related to the sexuality of patients indicate that sexuality has a broader meaning than the sexual act itself and that it is impossible to observe it outside the context of the overall development of the individual. Although definitions of sexuality include concepts such as self-awareness, self-esteem, body image, emotions, intimacy, gender, and sexual identity, first-year students mostly view sexual topics related to patient health from the perspective of the medical model through the concepts of sexual diseases, protection and reproductive health of young people.

*“Communication with patients about sexual health and sexuality, in general, I mostly associate with the prevention of sexually transmitted diseases…that is…sexual health of young people…”.*

The shift from a biomedical to a more holistic approach to patient care was evident across the years of study. Second-year students described sexuality and health primarily in terms of body-related satisfaction and self-expression: “*For me, sexuality means feeling good in my own body and being able to express myself.*” Third-year students focused on relationships and sexual orientation in the context of identity development: “*At this stage, sexuality is about relationships between men and women and about discovering your own sexual identity.*” Fourth-year students emphasised psychological and physiological needs and individual experience: “*Sexuality is a psychological and physical need, and it plays a role in recovery; everyone experiences it differently.*” Master’s students highlighted personal preferences and sexual enjoyment: “*Sexuality also includes personal preferences and sexual enjoyment.*”

**Perception of sexuality in the context of the patient’s psychophysical recovery**

The uniformity of the students’ statements regarding the appreciation of given topics in the context of the psychophysical recovery of patients is evident. Despite the verbalised discomfort when initiating topics related to the patient’s sexuality, students emphasise the necessity of education and training, especially in situations of postpartum recovery, reproductive complications (second-year students), as well as cerebrovascular insult and stoma (fourth-year students). The point of awareness is a significant motivator for starting a patient’s sexual health assessment, despite the personal discomfort, where it is important to include all patients, including those who do not report potential problems or concerns.

*“I am sure that sexuality is not the only topic that patients are reluctant to talk about, and they wish to be asked that kind of question so that they can open up and express their opinion, so nurses must be the first to start the discussion if they believe that the patient does not want to initiate.”*

In general, nursing students are aware of their role in educating and supporting the patient regarding sexual health, but are hesitant to take a proactive approach. First- and second-year students are guided by the comments, “*The practice of that type should be normalised*”. At the same time, in the final years of study, the concretisation of proactivity is observed in the sense of, “*What can I do to make a given set of questions a default part of professional practice?*”

*“I think that in addition to discussing the topic of sexuality with healthcare professionals, it would be good to know where to refer patients so that they can get additional information; because of that, it is important to define goals at the beginning, whether the purpose of communication is collecting data or providing information to patients, in order to write a nursing plan”* (master’s student).

#### 3.3.2. Theme 2: Inclusion of Sexual Health and Sexuality in Nursing Practice

**Obstacles during implementation**

For most students, a significant obstacle is a lack of knowledge, skills, and current information, which can lead to feelings of shame, discomfort, and, consequently, inadequate preparation for clinical practice. The student’s narrative emphasises the absence of sexuality topics in formal education, theory, and practice, which would make them more confident and question their assessment of incompetence and overwhelming fears.

*“I need adequate knowledge and theory…but also practice.” Despite all that, I need self-confidence and freedom of speech for that”* (second-year student).

Most nursing students across all study years report resistance when discussing topics related to patients’ ages, stating that they would feel most comfortable interacting with patients aged 18 to 30. Communication with elderly people is described as “difficult” and “unpleasant” because:

*“We do not think about the same things because we are not the same generation.” Maybe their time was different; maybe they did not have enough security and freedom…”* (third-year student).

Nursing students would take an avoidant position in discussing sexuality with oncology patients (first-year students) and terminally ill patients (second-year students). In addition, they believe that communication with people with mental disabilities would be a challenge due to additional pressure, which is reflected in the statements:

*“I think that some people with mental disabilities have different sexual preferences, which can lead to risky sexual behaviour, not only in terms of using protective measures but perhaps some oddities in the act itself, which would make communication difficult”* (second-year student).

Most nursing students do not express concern and uncertainty when discussing sexuality issues with patients of the opposite sex. In contrast, a smaller number of second and third-year nursing students fear that raising sexual health topics with people of the opposite sex would increase the risk of miscommunication, especially if the patient is male.

*“I wonder if someone who has problems with sexuality might misunderstand me… or, in fact, he/she would just like to get help”* (second-year student).

Although first- and second-year students express their fear of the reaction of older and more experienced colleagues to the opening of sexual topics, they believe that “*most medical staff would question whether it is really necessary*” and that “*older colleagues do not like to introduce new things…*”, but they believe that younger generations are “more open about it.” The change in narrative is evident in the responses of final-year students who verbalise the growing awareness of the potential problems of discrediting the nursing profession if sexuality is excluded from clinical practice because “*Information given by nurses is more valid than what he heard from a friend or on a TV show*”.

#### 3.3.3. Suggested Improvements

Although nursing students appreciate the multidisciplinary approach, a smaller number of them believe that the opening discussion of a patient’s sexual health is undertaken on the mutual responsibility of the patient and nurse, considering that *“The nurse should initiate, because it is important, for the treatment or generally for the patient’s health”.* In most students’ responses, they uniformly articulate the belief that nurses are the first choice of patients and consider such practice necessary, given that:

*“Nurses are most in contact with the patient, both during admission to the hospital and during treatment…the relationships they have with patients are more intimate”* (second-year student).

Students often suggest that their dilemma is determining how to start a conversation with a patient, unrelated to the difficulty of the topic (e.g., does the person have some form of disability), and that, at the same time, *“the patient does not interpret that I am attacking or hurting him”* (third-year student). Students recognise the usefulness of written educational material not as an alternative but as a complement to open communication. The nurses’ self-confidence would be enhanced by the presence of written guidelines or pre-prepared protocols, which would provide a structure and, as such, would be easily incorporated into the regular taking of nursing histories.

*“We need guidelines in the conversation, the questions we should most often ask, but also guidelines for sensitive groups”* (third-year student).

Additionally, students across all years of study emphasise the importance of privacy, confidentiality, and a non-judgmental approach by medical nurses to encourage, support, and educate patients.

An interesting observation is made by a master’s student, who positively assesses personal readiness to initiate such topics but questions the systemic incorporation and division of responsibilities with other health professionals, in the sense of “*What to do with the information obtained?”* and “*To whom should the patient be referred?”*, suggesting the necessary distribution of responsibilities, among all participants in the healthcare process, instead of delegating requests to one or a few practitioners.

Given that the majority of students report a lack of practical experience, which hinders their ability to express opinions on these topics, they believe that educational interventions can initiate changes in future practice, both through formal academic education and subsequent professional training. “*Ideal academic education would include general topics on sexuality, then sexuality in non-normal and atypical patients, sexuality in specific populations, equality and mental health in the context of sexuality, and finally sexual pathology.”*

*“The faculty provides information about the development of sexuality from anatomical, physiological, and histological aspects. However, that is one part of theoretical knowledge. The second part is how the students will access that communication … that is why the ideal education would focus on the physiology of everything, I think that is the basis, and then on the communication skills part”.*

Students state the need for additional lectures, workshops, or experiential learning concerning the specifics of patients, types of disabilities/disorders, and assistive technology that enables increasing sexual pleasure, and they hope that this will be possible for them in the continuation of their education.

## 4. Discussion

This study offers valuable insights into Serbian nursing students’ readiness to address sexual health issues in clinical practice. The validated Serbian version of the SA-SH-Ext demonstrated strong psychometric properties, with satisfactory composite reliability and acceptable convergent validity across factors, further supporting the stability of the identified factor structure, highlighting key dimensions that influence students’ attitudes and confidence, including *Being Comfortable, Communication with People with Disabilities, Future Patient and Working Relations*, and *Education and Competence*. These dimensions aligned closely with both prior research and the themes identified in our qualitative analysis. Despite an initially unsatisfactory model fit, exploratory factor analysis refined the structure, aligning with previous research on the importance of educational interventions in sexual health communication [14,31]. Nevertheless, the removal of several items and the marginally acceptable model fit indices indicate that the proposed factor structure should be interpreted with caution, and further validation in larger and more diverse samples is needed to confirm the robustness of the scale.

Despite generally positive attitudes, students reported significant discomfort in initiating conversations, uncertainty about their competencies, and fear of negative patient reactions. These concerns align with findings from Aaberg et al.’s study [4], suggesting that sociocultural beliefs, lack of education, and perceived patient resistance hinder open dialogue on sexual health.

A notable generational trend emerged: younger students expressed more openness but lacked experience, while senior students showed increased awareness but also hesitated due to entrenched norms and insufficient training [11]. These dynamics underscore the need for longitudinal educational strategies that begin early in nursing programs and intensify as the program progresses.

Cultural and social factors play a crucial role in shaping attitudes toward sexual health. Serbian students recognise its importance but struggle with conservative norms and insufficient training. Similar trends exist globally, where cultural values significantly influence professional attitudes toward discussions of sexual health [32,33].

A key finding was the need for formal sexual health education in nursing curricula. Serbian students reported receiving less training than those in Scandinavian countries, where such education has been integrated since the 1970s [33]. The lack of clear guidelines also contributes to discomfort, reinforcing the WHO’s recommendations for standardised sexual health education [34,35].

Interviews revealed specific concerns regarding discussions with individuals with disabilities and terminally ill patients. Despite existing guidelines, healthcare professionals often avoid addressing sexual health due to discomfort or perceived irrelevance [34]. Given the increasing prevalence of sexual dysfunction-related comorbidities, specialised training is essential.

The SA-SH-Ext subscales align with key themes from the interviews. *Being Comfortable* correlates with social stigma, emphasising the need for educational programs that promote open discussions. *Communication with People with Disabilities* reflects students’ uncertainty, highlighting the necessity of specialised training. *Future Patient and Working Relations* underscore fears of negative reactions, which may discourage addressing sexual health, while *Education and Competencies* reaffirm the need for curriculum improvements to enhance knowledge and practical skills.

Nursing curricula should incorporate communication training focused on sensitive topics to address these gaps. Courses should cover strategies for diverse patient populations, adaptive communication techniques, and the management of discomfort in clinical settings. Educational interventions should be tailored to generational differences, ensuring younger students gain professional communication skills while senior students receive intensive training to broaden their perspectives.

Overall, these findings underscore the importance of integrating sexual health education into nursing programs, thereby equipping students with the skills and confidence to engage in meaningful discussions. Future research should assess the long-term impact of educational interventions on students’ competencies in addressing sexual health.

### Limitations of the Study

This study has several limitations that should be considered when interpreting the findings. First, measurement invariance across subgroups (e.g., gender and year of study) was not formally tested. Although such analyses could further strengthen conclusions regarding the stability of the factor structure, subgroup sample sizes—particularly for male and master’s students—were insufficient for reliable multi-group confirmatory factor analysis. Future studies with larger and more balanced samples are needed to examine measurement invariance and confirm the stability of the scale across demographic and educational subgroups.

Second, the sample was drawn from a single institution, which limits the generalizability of the results to other regions or educational systems. In addition, the obtained model fit indices indicated only a marginally acceptable fit, suggesting that the proposed factor structure may not fully capture the underlying construct. Accordingly, future research should explore alternative model specifications and validate the factor structure using independent and more diverse samples.

Third, the qualitative data were collected through semi-structured interviews, which may be subject to social desirability bias, as students might have provided socially acceptable rather than fully candid responses. Although participants were informed that the interviewer was a university professor who was not involved in their teaching or assessment and held no formal academic authority over them, the interviewer’s academic role may nevertheless have influenced participants’ responses. This influence may have been further reinforced by the sensitive nature of sexuality-related topics and by prevailing cultural norms regarding sexuality within Serbian society.

Furthermore, the use of convenience sampling for the quantitative component and purposive sampling for the qualitative interviews may have limited the representativeness of the sample. Students who felt more open or comfortable discussing sexuality may have been more likely to participate, potentially contributing to generally positive attitudes and higher scores on the “Being Comfortable” dimension of the SA-SH-Ext questionnaire. Consequently, subgroup patterns and findings related to students’ attitudinal readiness to address sexual health should be interpreted with caution. Additionally, the interpretation of qualitative data may be influenced by researcher bias.

Finally, while the qualitative findings provide valuable insights into students’ attitudes and experiences, the relatively small sample size limits the generalizability of these results. Future longitudinal studies could examine changes in attitudes over time, particularly following targeted educational interventions. Assessing the impact of educational programs and staff training may offer further insight into improving clinical outcomes, enhancing patient satisfaction, and reducing stigma related to sexual health.

## 5. Conclusions

This study confirmed the reliability and validity of the Serbian version of the SA-SH-Ext questionnaire, identifying four key dimensions: *Being Comfortable, Communication with People with Disabilities, Future Patient and Working Relations*, and *Education and Competence*. The findings highlight significant barriers, including fear of negative reactions and lack of practical skills, which hinder nursing students from addressing sexual health in their future practice.

### Relevance or Clinical Practice

The findings of this study provide important implications for clinical nursing practice. By confirming the validity and reliability of the Serbian version of the SA-SH-Ext, healthcare educators and practitioners now have a robust tool for assessing nursing students’ readiness to address sexual health. Early identification of barriers such as discomfort, fear of negative patient reactions, and limited competence enables targeted educational interventions that can improve communication skills and enhance patient-centred care. Integrating structured sexual health training into nursing curricula and continuous professional development is crucial to reduce stigma, strengthen confidence, and prepare nurses to proactively address sensitive issues with diverse patient populations, including individuals with disabilities or chronic illnesses. Ultimately, enhancing nurses’ competence in sexual health communication contributes to holistic care, improved health outcomes, and increased patient satisfaction. The findings underscore the need for structured educational strategies, such as skills-based training, guided reflection, and supervised clinical discussions, to support nursing students in translating attitudinal readiness into competent sexual health practice.

## Figures and Tables

**Table 1:** Factor loadings, uniqueness, mean, and SD of the SA-SH-Ext.

		Factor					
	Items	1	2	3	4	Uniqueness	M (SD)
SA_SH_1	I feel comfortable about informing future patients about sexual health.	0.81				0.42	3.63 (0.97)
SA_SH_2	I feel comfortable about initiating a conversation regarding sexual health with future patients.	0.81				0.25	3.51 (0.97)
SA_SH_3	I feel comfortable about discussing sexual health with future patients.	0.89				0.16	3.73 (0.91)
SA_SH_4	I feel comfortable about discussing sexual health issues with future patients regardless of their sex.	0.74				0.21	3.68 (0.97)
SA_SH_5	I feel comfortable discussing sexual health issues with future clients with a physical disability.		0.65			0.31	3.43 (0.99)
SA_SH_6	I feel comfortable about discussing sexual health issues with future clients with physical diseases.		0.56			0.25	3.50 (0.98)
SA_SH_7	I feel comfortable about discussing sexual health issues with future clients with cognitive disability.		0.96			0.12	3.29 (1.01)
SA_SH_8	I feel comfortable about discussing sexual health issues with future clients with mental illness.		0.75			0.37	3.09 (1.01)
SA_SH_9	I feel comfortable about discussing sexual health issues with future patients regardless of their age.	0.55				0.48	3.51 (1.03)
SA_SH_11	I feel comfortable about discussing sexual health issues with future patients regardless of their sexual orientation.	0.55				0.48	3.78 (1.02)
SA_SH_12	I feel comfortable about discussing specific sexual activities with future patients.	0.78				0.43	3.29 (1.05)
SA_SH_15	I believe that future patients might feel embarrassed if I bring up sexual issues.			0.66		0.55	2.52 (0.83)
SA_SH_16	I am afraid that future patients might feel uneasy if I talk about sexual issues.			0.70		0.47	2.54 (0.83)
SA_SH_20	I am afraid that my future colleagues would feel uneasy if I brought up sexual issues with patients.			0.66		0.54	3.16 (0.98)
SA_SH_21	I am afraid that my future colleagues would feel uncomfortable in dealing with questions regarding patients’ sexual health.			0.75		0.42	3.11 (0.97)
SA_SH_24	I think that I, as a student, need to get basic knowledge about sexual health in my education.				0.63	0.62	4.44 (0.68)
SA_SH_25	I believe in my own ability to promote sexual health in my future profession.				0.72	0.39	3.78 (0.87)
SA_SH_26	I have sufficient competence to talk about sexual health with my future patients.				0.61	0.53	3.39 (0.91)
SA_SH_27	I think that I need to be trained in my education to talk about sexual health.				0.69	0.51	4.19 (0.81)

**Table 2:** Correlations between the SA-SH-Ext and SABS.

Subscale	Communication with People with Disabilities		Future Patient and Working Relations		Education and Competence		SABS	
	r	*p*	r	*p*	r	*p*	r	*p*
Being Comfortable	<0.001	<0.001	0.27	<0.001	0.44	<0.001	−0.50	<0.001
Communication with People with Disabilities	-	-	0.51	<0.001	0.21	<0.001	−0.47	<0.001
Future Patient and Working Relations			-	-	0.42	<0.001	−0.45	<0.001
Education and Competence					-	-	−0.53	<0.001

**Table 3:** Univariate analyses of the factors associated with Being Comfortable, Communication with People with Disabilities, Future Patient and Working Relations, and Education and Competence.

Variable	Category	Being Comfortable				Communication with People with Disabilities				Future Patient and Working Relations				Education and Competence			
		Mean	SD	t/F/NP	*p*	Mean	SD	t/F/NP	*p*	Mean	SD	t/F/NP	*p*	Mean	SD	t/F/NP	*p*
Gender	Female	41.70	9.28	2.24	0.02	18.45	3.87	0.92	0.35	12.42	2.52	0.27	0.78	22.51	3.08	0.99	0.32
	Male	46.30	9.15			19.26	4.16			12.26	2.92			23.21	3.66		
Study year	First	44.48	8.89	11.13	0.02	19.82	3.57	8.48	0.07	13.19	2.48	14.66	<0.001	22.84	3.10	3.55	0.47
	Second	41.55	9.31			17.80	3.77			12.36	2.43			22.77	3.52		
	Third	41.05	10.3			18.07	4.33			12.05	2.69			22.18	2.85		
	Fourth	38.86	7.41			18.40	3.71			12.18	2.50			22.22	3.10		
	Master	50.14	7.90			18.71	4.34			9.42	1.71			22.85	2.54		
Religious	Orthodox	41.99	9.24	1.33	0.51	18.25	3.90	5.68	0.05	12.29	2.47	1.80	0.40	22.65	2.98	1.26	0.53
	Catholic	44.84	8.11			20.76	2.80			12.38	3.01			23.00	3.46		
	Other	42.85	11.08			19.28	4.13			13.14	2.95			22.00	4.18		
Sexual orientation	Heterosexual	42.11	9.36	872.0	0.43	18.53	3.96	990.0	0.91	12.50	2.59	619.5	0.02	22.76	3.04	690.0	0.07
	Homosexual	44.91	9.50			18.53	3.12			10.91	1.83			20.41	4.14		
Total score	Whole sample	42.30	9.37			18.55	3.90			12.40	2.57			22.60	3.16		
t/F/NP—*t*-test, F-test or non-parametric replacement																	

## Data Availability

The datasets generated and analysed during the current study are available from the corresponding author upon reasonable request by qualified researchers. The data are not publicly available due to privacy and ethical reason.

## Public Involvement Statement

No public involvement in any aspect of this research.

## Guidelines and Standards Statement

The study adhered to the Consolidated Criteria for Reporting Qualitative Studies (COREQ) [20] and the Systematic Development of Standards for Mixed Methods Reporting in Rehabilitation Health Sciences Research (MMR-RHS) guidelines.

## Abbreviations

The following abbreviations are used in this manuscript:

SA-SH-Ext	Students’ Attitudes toward Addressing Sexual Health extended version
SABS	Sexual Attitude and Beliefs Survey
WHO	World Health Organisation
SA-SH	Students’ Attitudes toward Addressing Sexual Health
SA-SH-D	Students’ Attitudes toward Addressing Sexual Health Danish adaptation
COREQ	Consolidated Criteria for Reporting Qualitative Studies
MMR-RHS	Mixed Methods Reporting in Rehabilitation Health Sciences Research
SD	Standard deviation
ANOVA	One-way analysis of variance
LSD	Least Significant Difference
df	Degrees of freedom
CFI	Comparative fit index
SRMR	Standardised root mean square residual
RMSEA	Root Mean Square Error of Approximation
ICCs	Intraclass correlation coefficients
CIs	Confidence intervals
KMO	Kaiser–Meyer–Olkin
TLI	Tucker–Lewis Index

## Appendix A

**Table A1:** Interviewer characteristics and relationship with participants.

No.	Interviewer	Credentials	Occupation	Gender	Experience	Relationship Established	Participant’s Knowledge of the Interviewer	Interviewer Characteristics
**1**	NBJ	PhD	Psychologist, psychotherapist, professor	F	+	Some students knew that the interviewer was also a university professor	They have introduced the aim of the study	Interests in the research topic

**The symbol ‘+’ indicates that the interviewer has prior interviewing experience.**
